# The Sex Hormone Precursors Dehydroepiandrosterone (DHEA) and Its Sulfate Ester Form (DHEAS): Molecular Mechanisms and Actions on Human Body

**DOI:** 10.3390/ijms26178568

**Published:** 2025-09-03

**Authors:** Hsin-Yi Lin, Jie-Hong Chen, Kuo-Hu Chen

**Affiliations:** 1 Department of Obstetrics and Gynecology, Taipei Tzu-Chi Hospital, The Buddhist Tzu-Chi Medical Foundation, New Taipei City 23142, Taiwan; tuqiiko@gmail.com; 2 Department of Medicine, MacKay Medical University, New Taipei City 25245, Taiwan; albertjhc@gmail.com; 3 School of Medicine, Tzu-Chi University, Hualien 97004, Taiwan

**Keywords:** dehydroepiandrosterone, dehydroepiandrosterone sulfate, DHEA, DHEAS

## Abstract

Dehydroepiandrosterone (DHEA) and its sulfate ester form DHEAS, are multifunctional steroid hormones primarily produced in the adrenal cortex, with additional synthesis in peripheral tissues. DHEA/DHEAS serve as precursors to sex steroids and exhibit neuroprotective, anti-inflammatory, and immune-modulating effects. DHEA levels decline significantly with age, a phenomenon termed “adrenopause”, prompting interest in supplementation to mitigate age-related symptoms. Particularly in postmenopausal women, DHEA has shown potential benefits in treating genitourinary syndrome of menopause (GSM), including improved vaginal health, lubrication, and sexual function. While intravaginal DHEA appears effective and safer than systemic estrogen therapy, especially for women with estrogen sensitivity, results remain mixed for oral administration. DHEA and DHEAS exhibit diverse neuroactive properties through modulation of GABA-A, NMDA, and sigma-1 receptors. These neurosteroids contribute to neuroprotection, synaptic plasticity, and mood regulation. Altered DHEA/DHEAS levels have been implicated in neurodegenerative disorders and depression, with emerging evidence supporting their potential therapeutic value. In addition, DHEA plays a multifaceted role in aging-related physiological changes. It supports muscle anabolism, bone density maintenance, cardiovascular protection, and immune regulation. Though supplementation shows potential benefits, especially in conjunction with resistance training, results remain discrepant. Current evidence has revealed that the therapeutic effects of DHEA supplementation are inconsistent in different human systems among different studies. The diversity of results is mainly due to heterogeneous receptor distribution, various action pathways, and distinct tissue responses in different systems. Further research is needed to define its efficacy and dosage across various systems.

## 1. Introduction

Dehydroepiandrosterone (DHEA) was first isolated from human urine in 1934 by Butenandt and Dannenbaum [1], and its sulfated form, DHEAS, was discovered 10 years later by Munson, Gallagher, and Koch [2]. Its concentration decreases with age, leading to the hypothesis that restoring DHEA levels through supplementation may have anti-aging effects. DHEA was marketed as a “fountain of youth”, “super hormone”, “antidote for aging” or “mother of hormones”, and quickly introduced by the pharmaceutical industry in the 1980s, despite a lack of solid research supporting its benefits, and there were no large human studies until the 1990s. It was later banned as over-the-counter sales in 1985 by FDA [3] due to its lack of evidence of safety and efficacy. It was later approved by the Dietary Supplement Health and Education Act as a dietary supplement in 1994. However, the quality of DHEA and wide variety of content is somehow poorly regulated. The decline in DHEA/DHEAS levels with age [4] has been linked to the onset of many age-related chronic diseases and symptoms, such as osteopenia, sarcopenia, atherosclerosis, immunosenescence, and cognitive and mood impairment [3]. This has led to the hypothesis that restoring youthful DHEA/DHEAS levels through supplementation may provide health benefits in middle to older adults.

Dehydroepiandrosterone (DHEA) is a steroid hormone secreted by the adrenal gland, which is mainly secreted in Zona reticularis and is also found in other extra-adrenal organs such as gonads (ovarian theca cells and Leydig cells in the testes), the placenta, the liver, adipose tissue, and the central nervous system (CNS) [5,6]. Therefore, it is not only a sex hormone; it is also a neurosteroid. The ratio of dehydroepiandrosterone (DHEA) and its sulfated form as a way of storage, dehydroepiandrosterone sulfate (DHEAS), is 1:1000, and the circulating levels of both DHEA and DHEAS are influenced by sex and age, with women consistently exhibiting lower concentrations than men [7].

DHEA serves numerous biological functions, acting as an androgen, estrogen, and neurosteroid. DHEA and its sulfated form play diverse roles in the human body, which include neuroprotection, enhancing the growth of neurite, potential anti-cancer properties, antagonizing the oxidative stress, and metabolic, immune-modulating and anti-inflammatory effects. However, human clinical trials have produced inconclusive results that are unable to replicate these aforementioned findings consistently.

## 2. Method of Literature Review and Retrieval

The literature was searched to retrieve basic and clinical research, which investigated the molecular and cellular mechanism of DHEA/DHEAS production, conversion, and action, as well as the clinical associations (menopausal symptoms, effects in central nervous, metabolic, musculoskeletal, cardiovascular, and immune system) with DHEA and DHEAS actions. For the research topic, all studies were retrieved from these two databases Ovid Medline and PubMed using the search terms “dehydroepiandrosterone”, “dehydroepiandrosterone sulfate”, “DHEA”, and “DHEAS” in the current review. For screening and collection in the next stage, only English language with full-text articles were solicited for collection in a subsequent analysis.

In the next stage, duplicated articles were excluded. To ensure the quality of enrolled articles, two experts then inspected the articles to exclude studies with poorer study design, questionable methods or unclear research outcomes. Finally, a total of 141 articles were eligible for inclusion in this review.

## 3. The Molecular and Cellular Mechanism of DHEA/DHEAS Production, Conversion, and Action

**Figure 1:**
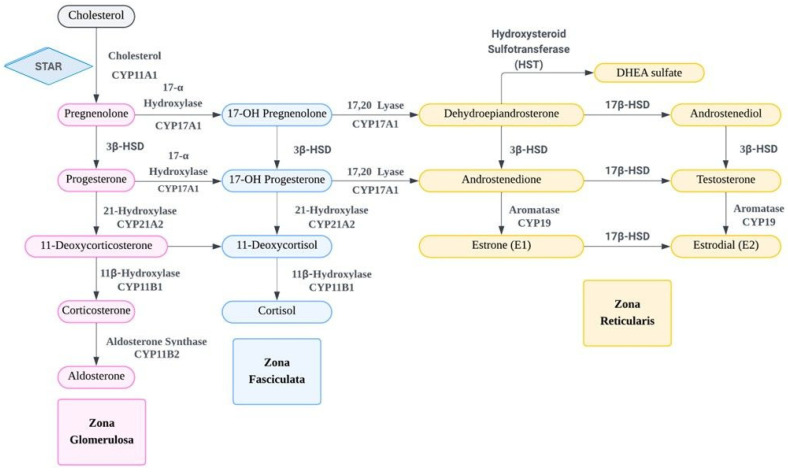
The synthesis and metabolism of dehydroepiandrosterone (DHEA)/dehydroepiandrosterone sulfate (DHEAS), adrenal hormones, and sex hormones. STAR: steroidogenic acute regulatory; HST: hydroxysteroid sulfotransferase; 3β-HSD: 3β-hydroxysteroid dehydrogenase; 17β-HSD: 17β-hydroxysteroid dehydrogenase.

DHEA, 5-androsten-3 beta-ol-17-one, and DHEAS belong to the C19 steroid family. Their biosynthesis in adrenal zona reticularis begins with the cleavage of cholesterol’s side chain by cytochrome P450scc (CYP11A1). CYP11A1 is anchored to the inner mitochondrial membrane by an amino-terminal α-helix. Its catalytic site faces the mitochondrial matrix. The transport of cholesterol from the outer mitochondrial membrane to the CYP11A1 catalytic site is facilitated by the steroidogenic acute regulatory protein (STAR, STARD1) and is converted into pregnenolone [8]. Pregnenolone produced in the mitochondria is transported to the endoplasmic reticulum. It is then hydroxylated to 17-hydroxypregnenolone and subsequently converted to DHEA by cytochrome P450c17 (CYP17A1) [9]. CYP17A1 is a type 1 integral membrane protein with two distinct catalytic actions. P450c17 (CYP17A1) is also expressed in the brain, where it may synthesize DHEA from pregnenolone. DHEA is converted into its more stable, hydrophilic sulfate ester, DHEAS, through sulfation. The enzyme is catalyzed by hydroxysteroid sulfotransferase (HST), also referred to as sulfotransferase family 2A member 1 (SULT2A1) or DHEA sulfotransferase [10]. It adds a sulfuryl (SO3) group to the C3-β-hydroxyl group of DHEA, producing DHEAS [11]. The synthesis and metabolism of DHEA/DHEAS are illustrated in Figure 1. DHEA is the active precursor for sex steroid synthesis and DHEAS does not bind to sex hormone-binding globulin (SHBG) [12], which also circulates freely in the blood. DHEA exhibits a shorter elimination half-life of 1 to 3 h [13] due to its weaker affinity to albumin, whereas DHEAS has a higher half-life of 13.7 h with its stronger albumin affinity and renal tubular reabsorption, leading to a relatively stable plasma concentration [14]. The clearance of DHEAS is approximately 13 L/day, significantly slower than the clearance of DHEA, which is around 2000 L/day [15].

**Figure 2:**
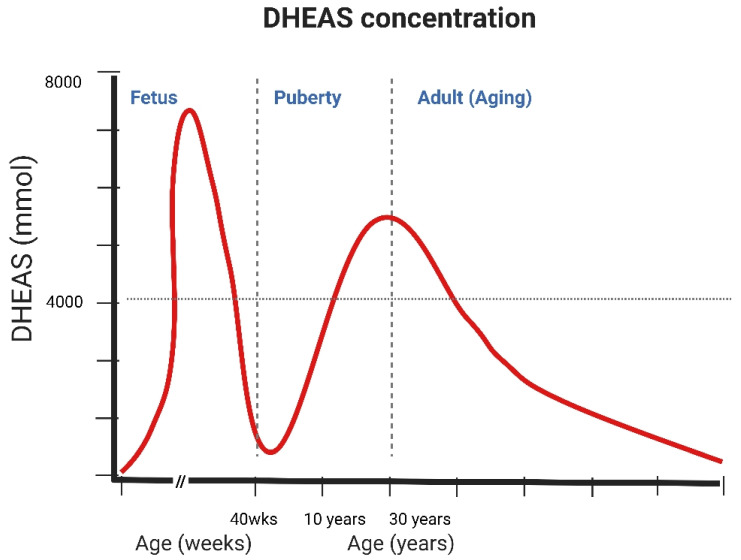
The fluctuation of DHEAS concentration with age.

DHEA and DHEAS plasma concentrations exhibit distinctive age-dependent patterns. During the fetal period, levels are remarkably high, ranging from 100 to 200 μg/dL (approximately 3–7 μM), but decline sharply postnatally and remain low until adrenarche, and between 7 and 10 years of age, the adrenal androgen production begins to rise [16]. Peak concentrations are reached between the ages of 20 and 30, with average DHEAS levels approaching 10 μM in men and 5 μM in women [17]. DHEA levels in young women are 10% to 30% lower than in young men. By age 70, DHEA/S levels are reduced to roughly 20% of their peak values, with potential declines up to 95% by ages 85–90 [7] (Figure 2). DHEA is the precursor for 30% to 50% of circulating androgens in older men and over 70% estrogen in older women [18]. This progressive reduction, in contrast to the relatively stable cortisol levels, has been termed as “adrenopause,” highlighting a specific aspect of endocrine aging characterized by diminished adrenal androgen secretion [19]. One proposed mechanism for the age-related decline in DHEA and DHEAS involves reduced activity of either the enzyme 17-alpha-hydroxyprogesterone aldolase, which catalyzes the conversion of pregnenolone to 17-hydroxypregnenolone, or enzyme 17,20-lyase, which catalyzes the conversion of 17-hydroxypregnenolone to DHEA or in the adrenal gland [20].

The role of DHEA/DHEAS in peripheral sex hormone production is particularly significant in women. While testicular androgen production in men remains relatively stable throughout life, ovarian estrogen production drops sharply and ceases at menopause, which then totally depends on adrenal gland production of sex hormones. This emphasizes the importance of intracorporeal DHEA/DHEAS in its role of maintaining estrogenic activity during the postmenopausal stage [21]. During menopausal transition, a substantial amount reveals an increase in DHEA, which was suspected to be a potentially compensatory elevation in adrenal androgen secretion. Studies show ethnic differences in DHEA levels during menopause, while Caucasians and women of Chinese ethnicity exhibit the fastest decline, and African-American women have the lowest overall levels [22].

**Figure 3:**
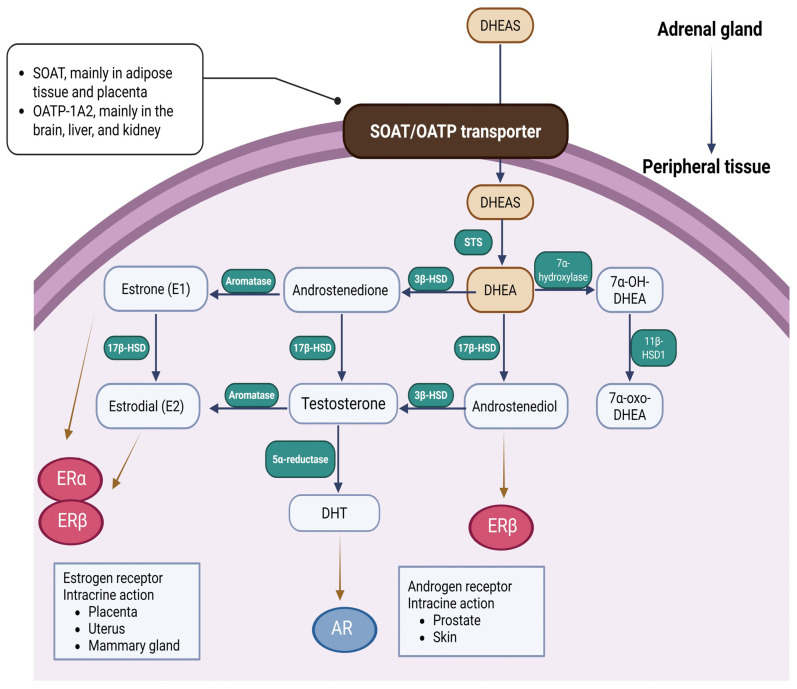
The transportation and utilization of DHEA/DHEAS from adrenal gland to peripheral tissues. SOAT: solute organic anion transporter; OATP: organic anion transporter polypeptide; 3β-HSD: 3β-hydroxysteroid dehydrogenase; 17β-HSD: 17β-hydroxysteroid dehydrogenase; DHT: dihydrotestosterone; STS: steroid sulfatase; ER: estrogen receptor; AR: androgen receptor.

Figure 3 illustrates the transportation and utilization of DHEA/DHEAS from adrenal gland to peripheral tissues. Transportation of peripheral DHEAS predominantly depends on solute organic anion transporter (SOAT, mainly in adipose tissue and placenta) and organic anion transporter polypeptide-1A2 (OATP-1A2, mainly in the brain, liver, and kidney). In the peripheral tissues, cells that express steroid sulfatase (STS) can convert DHEAS into DHEA, which can later be further metabolized into sex hormones in specific cell types [23]. This localized, cell-specific production of androgens and estrogens is known as an intracrine mechanism of action [24].

DHEA is later converted to androstenedione (4-androstene-3β,17β-dione, Adione) by 3β-hydroxysteroid dehydrogenase (3β-HSD) and androstenediol (5-androstene3β,17β-diol, Adiol) by 17β-hydroxysteroid dehydrogenase (17β-HSD) [23]. Androstenedione (Adione) is converted to testosterone by 17β-hydroxysteroid dehydrogenase. Testosterone is then converted to dihydrotestosterone (DHT), the most potent androgen via 5-alpha reduction. DHT can be further metabolized to androstane-3α, 17β-diol (3-diol). 3-diol can then undergo glucuronidation, producing 3-diol-3-glucuronide (3-diol,3G) and the predominant peripheral androgen marker, 3-diol-17-glucuronide (3-diol,17G) [25]. 3-diol, 17G in plasma serves as a biomarker of peripheral androgen activity, and its significant elevation has been linked to idiopathic hirsutism [26]. DHEA can be converted to estrone (E1) and estradiol (E2) through its intermediate forms, androstenedione (Adione) and testosterone, respectively, via the action of aromatase. Aromatase is a key enzyme in sex hormone synthesis, converting androstenedione to estrone (E1) and testosterone to estradiol (E2) through aromatization. Estrone and estradiol can be interconverted by the enzyme 17β-HSD.

DHEA acts through multiple signaling pathways on hormone receptors, which include nuclear receptors, such as estrogen receptors α (ERα) and β (ERβ), androgen receptors (AR), peroxisome proliferator-activated receptors (PPARs), and pregnane X receptor/steroid and xenobiotic receptor (PXR/SXR, NR1I2), as well as membrane-associated signaling pathways via G protein-coupled receptors, which then subsequently activate MAPK and PI3K second messaging pathways, modulating neurotransmitter receptors N-methyl d-aspartate (NMDA) receptor, γ-aminobutyric-acid type A (GABAA), and sigma-1 receptors inhibition of voltage-gated T-type Ca^2+^ channels.

## 4. Clinical Associations with DHEA and DHEAS Actions

### 4.1. DHEA on Menopausal Symptoms

There were multiple studies suggesting that pharmacokinetics differences in DHEA on older men and on older women. Frye et al. conducted a single-blind, placebo-controlled trial with a single dose of DHEA and found the sex difference that DHEA concentrations increased 5- to 6-fold in both genders, while DHEAS levels rose 5-fold in men and 21-fold in women compared to baseline [27].

The hormonal fluctuations during the postmenopausal period can cause a variety of symptoms that significantly affect women’s life quality. Genitourinary syndrome of menopause (GSM) affects genital structures, including the labia, clitoris, vagina, urethra, and bladder [28]. Their symptoms typically include thinning, dryness, and inflammation of the vaginal epithelium, painful intercourse, and urinary issues such as urgency, frequency, dysuria, and incontinence. It is important to note that the female genitalia and lower urinary tract originate from the same embryological source and are sensitive to female sex steroid hormones, and that estrogen serves an imperative role to maintain the integrity in these tissues [29]. The vagina contains steroidogenic enzymes that can convert DHEA into estrogens. Estrogen plays a crucial role in maintaining the structure and function of the vaginal wall, including the squamous epithelium, lamina propria, and smooth muscle layer. In the lamina propria, estrogen promotes vasodilation, while estradiol (E2) maintains a dense, mature epithelial layer, supports vaginal smooth muscle, and enhances tissue elasticity [30]. Without estrogen, the vagina undergoes atrophic changes with dryness, itching, and less functional smooth muscle contraction that can negatively impact sexual function and satisfaction and increase susceptibility of infection [31].

While DHEA’s androgenic effects are achieved by being converted to testosterone to improve libido along with its estrogenic effects resulting in improvements in menopausal vasomotor symptoms, DHEA has been introduced to postmenopausal women in order to relieve their discomfort. Additional attention is also given to its effects on systemic organs, such as the bone, skin, muscle, vagina, adipose tissue, glucose parameters, and cognitive function after oral and percutaneous administration. After receiving DHEA through multiple routes, including oral, transdermal or vaginal routes, DHEA has been found to be mainly transformed into androgens rather than estrogens. In hypoadrenal women who have undergone natural or surgical menopause, androgens given in addition to estrogens have provided a beneficial effect on sexuality [32]. A previous study has shown that oral DHEA undergoes significant first-pass hepatic metabolism mainly by hepatic 5-reductase, which favors androgenic biotransformation by increasing downstream DHT metabolites such as 3-diol-3G, 3-diol-17G, and ADT-G to four-to-five-fold, whereas transdermal administration may only increase its metabolites by about 71% [33,34].

**Figure 4:**
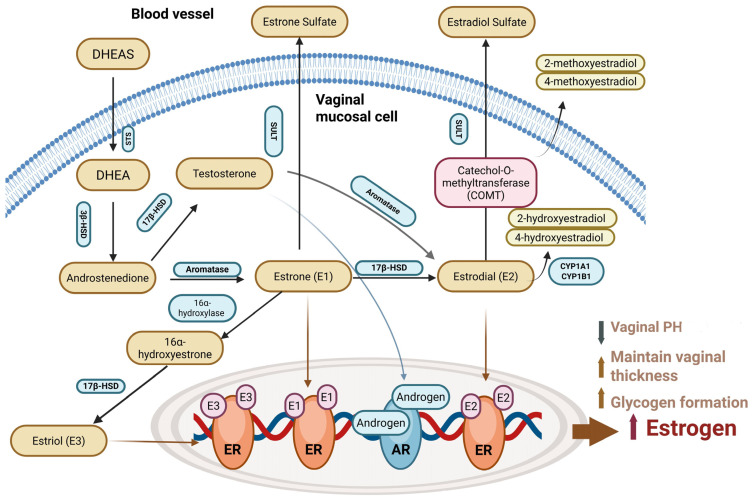
The detailed mechanism of intravaginal DHEA/DHEAS and estrogen usage. SULT: estrogen sulfotransferase.

Vaginal estrogen therapy is effective for GSM but may not be suitable for women who are suffering or predisposed to gynecologic or breast cancers, or using aromatase inhibitors (AIs), and this reason may lead to reluctance among women to seek treatment, causing them to endure discomfort and pain. Therefore, DHEA is introduced to avoid direct estrogen exposure. The detailed mechanism of intravaginal DHEA/DHEAS and estrogen usage is shown in Figure 4.

The absence of enzymes that are able to convert DHEA into estrogens in the endometrium explains the typical endometrial atrophy observed in all normal postmenopausal women, despite varying levels of circulating endogenous DHEA. According to this mechanism, it has been noticed that DHEA supplementation can stimulate the vagina exclusively and not the endometrium [35]. Multiple randomized controlled trials (RCTs) have accessed women with menopausal syndrome after being treated with daily vaginal DHEA for 12 weeks, and enhanced vaginal health was observed with improved epithelial maturation in cytology along with decreased PH level without increasing systemic estrogen levels [31]. In these trials, no “off target” effects were observed without any significant changes in osteocalcin or bone alkaline phosphatase. Significant improvement was noted in patients receiving DHEA, with effects measured by using Sexual Function Index (FSFI), which include subscales of sexual arousal, lubrication, pain, and satisfaction. A higher dosage of 6.5 mg DHEA administration revealed better improvement in sexual arousal and pain subscales in comparison to a dosage of 3.25 mg DHEA, and other studies showed that androgenic side effects such as voice change and headache were more commonly reported in women receiving DHEA [31,36,37]. While comparing the vaginal pap smears after application of vaginal DHEA, decrease in parabasal cells and increase in superficial cells were highly significant than those with placebo use at all time intervals (from 2 to 12 weeks interval), and the effect was not inferior to the effect of applying the vaginal E2 and conjugated estrogen formulations. When parabasal cells are predominant, it indicates hypoestrogenemia and atrophy. Promoting the transfer to more superficial, mature epithelial cells is the primary goal of treatment for relieving symptoms of vulvovaginal atrophy (VVA) [36]. Symptom-wide improvements were also observed particularly in cases of moderate to severe dyspareunia and vaginal dryness [35]. Nevertheless, while daily DHEA use significantly improved VVA symptoms, administering vaginal DHEA only twice per week did not show considerable benefit [38].

A recent systematic review concluded that vaginal DHEA therapy appeared to be more effective in perimenopausal or postmenopausal women than in other populations in aspects such as sexual interest, lubrication, pain, arousal, orgasm, and sexual frequency [39]. However, in women receiving oral DHEA supplementation, it was found that DHEA was associated with no significant improvement in libido and sexual function [30,40].

Postmenopausal women with mild to moderate urinary urgency were enrolled in a study comparing the effects of DHEA and hyaluronic acid over a 12-week period. The results showed a reduction in the severity of urinary urge incontinence [41] in all cases. A latest pilot study evaluating intravaginal DHEA usage and employing a 3-day bladder diary and the International Consultation on Incontinence Questionnaire showed that intravaginal DHEA significantly alleviated urinary symptoms, improved quality of life, and enhanced pelvic floor muscle function in postmenopausal women with stress urinary incontinence (SUI) and VVA [42].

### 4.2. DHEA in Central Nervous System

Myriad studies have established the importance of DHEA and DHEAS in the central nervous system. DHEA and DHEAS have numerous interactions with the receptors of the brain, which mainly include the γ-aminobutyric acid type A (GABA-A) receptor, the N-methyl-D-aspartate (NMDA) receptor, and the sigma-1 (σ1) receptor [43,44,45,46]. Other receptors such as microtubule-associated protein 2 (MAP2) [47], membrane DHEA binding sites (mDBS) [48], plasma membrane receptor tropomyosin receptor kinase (Trk)-A, and p75 neurotrophin receptor (p75NTR) [49] are also involved in their further interactions.

NMDA receptors, a type of glutamate receptor, play a complex role in neuroprotection. While excessive activation of NMDA receptors can lead to neuronal damage (excitotoxicity), particularly in conditions like stroke, they also have a protective role under normal and some pathological conditions. Overstimulation of NMDA receptors by glutamate can lead to excessive calcium influx into neurons, triggering a cascade of events that ultimately lead to cell death. In contrast, proper NMDA receptor activation can trigger a cascade of events that promote neuronal survival and protect against damage, such as activating antioxidant defenses and modulating ionic channels. NMDA receptor antagonists, while potentially neuroprotective in some contexts, can also impair normal neuronal function and cause side effects. In summary, NMDA receptors play a dual role in neuronal health, acting as both potential mediators of excitotoxicity and triggers for neuroprotective mechanisms. Targeting NMDA receptors for neuroprotection requires a careful understanding of the complex interplay between their excitatory and protective functions, as well as the potential for both beneficial and detrimental effects of receptor modulation.

DHEA and DHEAS are both non-competitive antagonists towards GABA-A receptors, whereas DHEAS demonstrates a more potent inhibitory tendency [50,51]. DHEAS also potentiates NMDA receptor function and modulates synaptic plasticity through its action as a σ1 agonist [10] which potentiates glutamatergic activity. But in non-hippocampal brain regions such as striatum, DHEAS has been found to inhibit NMDA-induced dopamine release [52]. A multitude of studies have suggested that DHEA and DHEAS enroll in brain development such as neuroprotection and stimulation of neurogenesis, and play a role in the regulation of apoptosis. The neuroplastic effects are mediated through their interactions with ionotropic amino acid receptors, GABA-A receptors, σ1 receptors, as well as the MAPK signaling pathway [53]. DHEA also exhibits potent antioxidant, anti-inflammatory, and anti-glucocorticoid properties while encountering stressful events [10]. DHEA can activate Akt kinase protein by promoting its phosphorylation, which later inhibits pro-apoptotic factors, thereby protecting neurons from oxidative stress, glutamate toxicity, and corticosterone [54].

**Figure 5:**
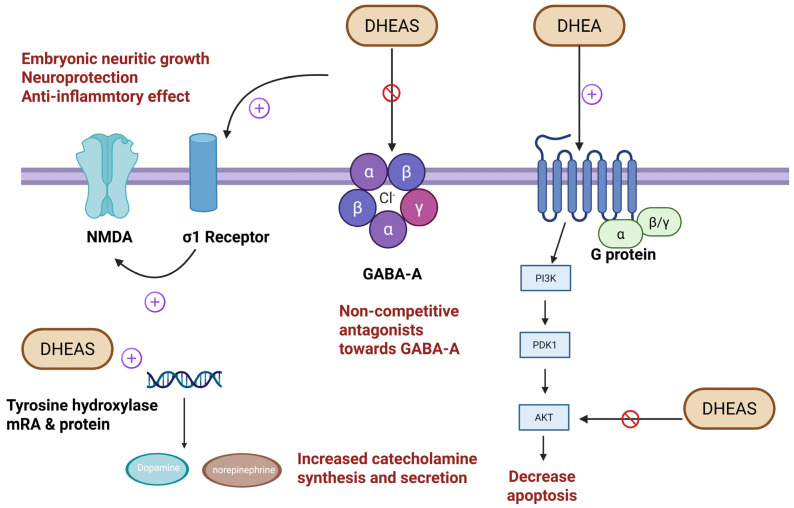
The actions of DHEA and DHEAS on the central nervous system via different membrane receptors. GABA-A receptor: γ-aminobutyric acid type A receptor; NMDA receptor: N-methyl-D-aspartate receptor; σ1 receptor: sigma-1 receptor.

The neuroprotective effect of DHEA against NMDA-related cytotoxicity is mediated via direct NMDA receptor modulation of the calcium–nitric oxide (Ca^2+^/NO) signaling pathway, whereas the protective effect of DHEAS may be mediated through activation of the sigma-1 (σ1) receptor. The sigma-1 receptor is a unique ligand-regulated molecular chaperone located in the endoplasmic reticulum and is expressed in the heart, kidney, liver, and brain [55,56]. On the other hand, the σ1 receptor antagonists rimcazole and 1-[2-(3,4-dichlorophenyl)-ethyl]-4-methylpiperazine (BD1063) partially but significantly reverse the protective effect of DHEAS against NMDA-related neurotoxicity [57]. Figure 5 depicts the actions of DHEA and DHEAS on the central nervous system via different membrane receptors.

DHEAS, but not DHEA, augments cholinergic function by acting as a GABA-A receptor inhibitor [58], and was found to prevent (in a dose-dependent manner) the memory impairment induced by the ACh receptor antagonist scopolamine in mice in a previous study [59]. Alzheimer’s disease (AD) is the most common cause of dementia, and it is a progressive neurodegenerative disorder characterized by the gradual decline in memory, cognitive function, language, and reasoning. Postmortem studies in patients with Alzheimer’s disease have revealed significantly reduced levels of DHEAS in the striatum, cerebellum, and hypothalamus and suggest that cognitive decline in Alzheimer’s disease may be more closely associated with reductions in DHEAS concentrations, or a decreased DHEAS-to-DHEA ratio, rather than absolute DHEA levels alone [60]. Previous studies have also found that plasma DHEAS concentrations are significantly lower in Alzheimer’s disease (AD) patients compared to healthy controls [61]. DHEAS protects neurons from oxidative stress β-amyloid toxicity [62] via the sigma-1 receptor. However, previous clinical trials in patients with Alzheimer’s disease have reported no significant cognitive benefits following DHEA supplementation [63].

The salival and serum DHEA-to-cortisol ratio, rather than individual hormone levels, has been shown to more accurately differentiate depressed from non-depressed individuals [64]. During acute stress episodes, steroid biosynthesis may be shifted from anabolic adrenal androgen pathway to catabolic corticosteroid pathways to ensure the energy of the mobilization to overcome the stressor. While comparing salivary DHEA, DHEAS, and cortisol/DHEA levels in response to acute psychosocial stress in patients with depressive disorders, lower salivary DHEA, elevated cortisol, and elevated cortisol/DHEA levels in the depression group were noted [65].

Estradiol plays a great role in the brain and through ERα and ERβ nuclear receptors, it regulates gene expression and induces long-term changes in synaptic and neural function. ERβ is predominant in brain regions such as the hippocampus and thalamus that are crucial for cognition. G protein-coupled estrogen receptors are found mainly in the prefrontal cortex and hippocampus, which are responsible for the acute effects of estrogen on cognition [66]. Estradiol’s interaction and modulation of the serotonergic and cholinergic systems are especially relevant to the development of depression. Therefore, fluctuation in estradiol, such as age-related declines in ovarian estrogen production after menopause [67] may affect the response of stress and emotional–cognitive processing, potentially influencing the risk of depression [68]. However, multiple studies have reported contradicting results with regard to the DHEA/DHEAS concentration in postmenopausal women with depression, where no association between DHEAS and depression was found in different cross-sectional studies, and some others discovered mixed results of depression severity [69,70].

A recent meta-analysis [71] of 14 studies has evaluated the effects of oral DHEA (25–400 mg daily for 6 weeks to 12 months) in patients diagnosed with depression. The findings suggest DHEA has been associated with beneficial effects on depressive symptoms when compared to placebo, and the therapeutic benefits are most evident in individuals with major depression, dysthymia, or those experiencing mild to moderate depressive symptoms. A recent study also suggests that higher circulating DHEA/DHEAS can predict SSRI-associated remission in major depression [72].

### 4.3. DHEA in Metabolic System

DHEA has been shown to improve diabetes mellitus and obesity in animal studies by reducing hyperglycemia, increasing insulin sensitivity, and preserving pancreatic beta cell function and structure [56]. Administration of DHEA in mice was considered to decrease gluconeogenesis in the liver by suppressing the gene expression and activity of liver glucose-6-phosphatase and enhancing uptake of 2-deoxyglucose in HepG2 cells [73]. It was also proposed that DHEA may activate the PI3K/Akt-PFK-2 signal pathway [74]. A study revealed that DHEA administration increased the activities of glycolytic enzymes, including hexokinase (HK), glucokinase (GK), and phosphofructokinase (PFK), in the muscle of db/db mice [75]. The other studies regarding DHEA in the human body showed diverse results. Phillips et al. found that the strongest association with metabolic syndrome was the cortisol-to-DHEA ratio, with a higher ratio indicating a greater risk of developing metabolic syndrome [76].

In premenopausal women, elevated serum DHEAS is associated with a higher amount of fat distribution on the trunk and lower amounts on the lower limbs, suggesting a central adiposity [77]. A meta-analysis has included elderly men receiving DHEA supplementation, and reduction in fat mass was observed, but multivariate analysis suggested that this effect might be more closely related to variations in circulating testosterone (TT) and estradiol (E2) levels than to DHEA *per se*. However, DHEA showed no significant effects on glycemia, insulin, total cholesterol (TC), or bone mineral density (BMD) compared to placebo [78]. Also, there was no significant effect on serum lipids (including LDL, HDL, Triglyceride), glucose level, or BMD (except for a small change in lumbar spine BMD) in another meta-analysis evaluating postmenopausal women [40]. In a randomized double-blind trial with SLE patients, DHEA administration led to reductions in triglycerides and HDL levels. This suggests that androgens may increase hepatic lipase activity which later enhance reverse cholesterol transport by promoting HDL clearance, rather than reducing HDL production.

Aoki et al. conducted research to establish whether DHEA/DHEAS or its metabolites (such as androstenedione) are responsible for improving hyperglycemia, and androstenedione was administered to C57BL6 mice on a high-fat diet. The result showed androstenedione did not increase Akt phosphorylation in the liver, and it only lightly reduced blood glucose levels compared with DHEA, which further supports the idea that the hypoglycemic effects of DHEA are primarily mediated by DHEA or DHEAS *per se*. Studies on the relationship of DHEA and insulin sensitivity, however, reported mixed results. The possible mechanisms behind the improvement in diabetes mellitus may include increased glucose uptake in adipocytes by promoting the translocation of GLUT4 and GLUT1 through the plasma membrane, antioxidative effects, and preventing production of advanced glycation end-products. Diabetic patients exert chronic inflammation leading to the release of proinflammatory cytokines and macrophage activation, which can contribute to insulin resistance. DHEA supplements may decrease inflammation by inhibiting plasma IL-6 and TNF-alpha. A recent meta-analysis has concluded that DHEA supplement can lower fasting glucose levels; however, there are no effects of DHEA supplementation on insulin levels and the homeostasis model assessment-estimated insulin resistance (HOMA-IR) index in humans [79].

### 4.4. DHEA in Musculoskeletal System

The progressive decline in muscle mass and strength is a common issue with aging in humans, possibly due to a gradual shift from anabolic to catabolic status. Skeletal muscle is a sex steroid-sensitive tissue capable of locally synthesizing 5α-dihydrotestosterone (DHT) from either testosterone or DHEA. Since DHEA is a precursor of testosterone, DHEA supplementation has been proposed to be a potential intervention to increase muscle strength, preserve muscle mass, and decrease bone loss. To counteract this decline, DHEA supplementation has been proposed to maintain levels within the “youthful” range. Another possibility is that DHEA replacement might enhance muscle anabolism by increasing IGF-I concentration [80] and the mechano-growth factor isoform during muscle contraction [81], along with its anti-glucocorticosteroid effects [82].

Several RCTs were conducted on mid-to-old age women, and after receiving 50 mg DHEA supplementation for 12 weeks, the result did not reveal any improvement in muscle mass [83], function, or endurance. A recent meta-analysis included nine publications with elderly women over 60 years old and found DHEA supplementation resulted in increased testosterone levels and lean body mass with decreased fat mass, BMI, and body weight [84]. The result further supported that testosterone could reduce visceral fat. While DHEA replacement alone did not significantly increase muscle mass or strength in elderly men and women, DHEA replacement significantly enhanced the effects of heavy resistance exercise on muscle hypertrophy and strength in elderly women and men [85]. Aging is associated with chronic elevation of inflammatory cytokines, IL-6, TNF-α, and C-reactive protein (CRP) [86]. DHEAS is a specific activator of peroxisome proliferator-activated receptor-alpha (PPARα), which antagonizes signaling through pathways such as STATs, AP-1, and NF-κB. This reduces inflammation and helps counteract the catabolic effects on skeletal muscle [87]. During weight training, DHEA can reduce the inflammatory cytokine response and enhance the serum testosterone and androgenic effects, which may, in turn, promote muscle mass gain during resistance exercise.

Osteopenia and osteoporosis are significant medical concerns, as they increase the risk of fractures and disability. Aging is associated with decline in bone mineral density (BMD). Previous studies have shown that the peak and nadir of BMD consistently correspond to serum DHEA, with low DHEA being linked to reduced BMD and an increased risk of osteoporotic fractures [88]. Previous research on animal models has shown DHEA possesses the ability to increase BMD on rats with osteoporosis, and the bone formation is mainly mediated by the DHEA-derived androgens [89].

A recent meta-analysis including several RCTs focusing on elderly patients has shown DHEA replacement can increase BMD of hip and trochanter in women [18]. Serum bone-specific alkaline phosphatase in patients receiving DHEA is lower, suggesting attenuation of bone turnover. This effect is mainly linked to its estrogenic effect on the bone, which helps inhibit bone resorption by reducing osteoclast activity and promoting osteoblast activation, leading to a positive effect on bone density [90]. Osteoclasts in the bone multicellular unit (BMU) promote bone resorption and enhance the hydroxyapatite mineral layer dissolution and enzymatic degradation of the bone protein matrix. In contrast, osteoblasts in BMU form the bone matrix by depositing unmineralized tissue called osteoid, which then undergoes a regulated mineralization process including proliferation, migration, and activation and later forms bone tissue [10,91]. DHEA replacement has also been linked to increased IGF-1 levels, which can enhance anabolic effects on the bone, leading to bone formation and reduced bone loss [92]. Another study has also recognized DHEA as a targeted therapy for elderly women to enhance their lumbar spine and hip BMD, which could be a possible strategy to slow down the decline of BMD in postmenopausal women who are not candidates for conventional osteoporosis treatments [93].

### 4.5. DHEA in Cardiovascular System

A bunch of research has shown that DHEA inhibits platelet aggregation [94], increases fibrinolysis [95], reduces atherosclerosis, and lowers cholesterol in the coronary and aortic arteries [96]. Likewise, recent findings have suggested that DHEAS can increase proliferation, enhance angiogenesis, and inhibit apoptosis; hence, its absence may elevate the risk of inflammation and damage of the endothelium and has been linked to an increased risk of cardiovascular disease [56,97,98,99,100]. A significant inverse association between DHEA levels and coronary artery calcification (CAC) on CT scans has been observed in elderly men. The relationships between DHEA/testosterone and CAC appear to be at least partially independent, suggesting that adrenal and testicular androgens may each contribute independently to cardiovascular health [101].

In the cardiovascular system, DHEA exerts its beneficial effects by modulating several key processes, including membrane potential, ion channel function, endothelial nitric oxide (NO) production, oxidative stress, cell proliferation, apoptosis, and various signaling pathways. DHEA’s membrane receptor signaling comes from bovine studies demonstrating high-affinity binding to membrane-associated receptors in the aorta, heart, and liver [56]. DHEA can stimulate GTPγS binding to Giα2 and Giα3 proteins in endothelial cells, and this effect can be blocked by antibodies targeting these G proteins. DHEA binding to these receptors increases NO synthesis [102] in the endothelial cells, which subsequently leads to increased cGMP production in smooth muscle cells (SMCs), which is an imperative cellular component in blood vessels, causing relaxation. This effect is independent from androgen and estrogen receptors. DHEA was shown to inhibit voltage-dependent T-type Ca^2+^ channels by a Gi protein-dependent pathway [103], reduce intracellular calcium by upregulating potassium channels, and promote SMC relaxation [56]. In systemic circulation, DHEA has also been shown to inhibit the Akt/GSK-3β signaling pathway in vascular remodeling, reducing cell proliferation and selectively inducing apoptosis in proliferating cells [104]. DHEA stimulates the sigma-1 receptor (σ1R), activating the Akt-eNOS signaling pathway, which promotes vascular protection. These combined effects reduce SMC proliferation and arterial remodeling, providing potential cardiovascular benefits.

Low levels of DHEA were found to be a poor prognostic factor in patients with cardiovascular diseases in a meta-analysis enrolling all English-language and Chinese-language published studies [105]. Jia et al. found that low levels of DHEAS are associated with subclinical myocardial injury, heart failure (HF) hospitalization, and death. They also observed a non-linear relationship, with a threshold below which very low DHEAS levels significantly increased the risk of HF and mortality, while higher levels did not provide additional protective effects [106]. Among postmenopausal women undergoing coronary angiography, lower DHEAS levels were associated with higher mortality both in CVD and all-cause mortality [97]. There was a connection between low levels of DHEA and lower right ventricular function, and it was suggested that supplementation of DHEA could possibly reverse cardiac hypertrophy and enhance tissue levels of collagen and formation of fibronectin, which subsequently decrease myocardial fibrosis as a consequence of right ventricle remodeling and pulmonary arterial hypertension decompensation [30,107,108].

### 4.6. DHEA in Immune System

With aging, the function of the T-helper 1 (Th1) cell is impaired with a reduced ability to activate antibody production by CD5− B cells. Certain immune responses may decline, such as antibody responses to vaccines, T cell proliferation, and interleukin-2 (IL-2) production. In contrast, other immune activities increase, including the production of autoantibodies and cytokines like IL-4, IL-5, IL-6, IL-10, and interferon-gamma (IFN-γ). The relationship between the immune system and neuroendocrine systems is closely interconnected. Partial dysfunction of the hypothalamic–pituitary–adrenal (HPA) axis is noted in chronic inflammatory diseases [109], where at first a spike of both cortisol and DHEA occurs after a proinflammatory stimulus in response to increased ACTH [110]. Nonetheless, after repeated stimulation, this acute response becomes altered, potentially leading to adaptive down-regulation of the HPA axis, markedly affecting the production of androgens. Adrenal steroids can influence immune cells and impact the progression of autoimmune diseases. The loss of adrenal androgen precursors results in a significant decrease in peripheral sex hormone levels. This is especially important in postmenopausal women and elderly men, as adrenal androgens become the main source of sex hormones due to the natural decline in gonadal steroid production with aging [109]. During acute inflammation, proinflammatory cytokines such as IL-1B, TNF-α, and IL-6 stimulate the release of corticotropin-releasing hormone (CRH) from the hypothalamus, which in turn increases the secretion of adrenal hormones, including cortisol and DHEA [111,112]. In previous animal models, DHEA supplementation has been shown to improve arthritis [113]. DHEA possesses anti-inflammatory and immunomodulatory properties, which include inhibitions of proinflammatory cytokine production by blocking NF-κB activation and AP-1 inhibitor, enhancing the ratio of Th1/Th2 cytokine production and anti-glucocorticoid properties [109].

Low levels of DHEA have been observed in autoimmune diseases such as systemic lupus erythematosus (SLE), rheumatoid arthritis (RA), and Sjögren’s syndrome (SS). In SLE, autologous cortisol and DHEA are significantly reduced by around 50%, especially in the active phase. This reduction is further exacerbated by receiving corticosteroid treatment, which suppresses ACTH secretion through negative feedback, leading to a subsequent decrease in DHEA production. Patients with SLE express a cytokine imbalance attributed to a defect in CD4+ Th1 cells or inhibition of Th1 cells by excessive production of IL-6 and IL-10 [114]. In vitro, DHEA decreases proinflammatory cytokines IL-6 and increases IL-2 expression. In animal studies, DHEA delays the production of double-stranded DNA antibodies and enhances the survival rate [114].

Previous studies evaluating the effectiveness of DHEA in SLE have been challenging due to its multi-organ involvement and its episodic exacerbations, which complicate the assessment of consistent outcomes. DHEA has shown a modest but clinically meaningful improvement in health-related quality of life over the short term in previous RCT studies [115]. A randomized, double-blind placebo-controlled trial enrolled mild-to-moderate patients taking standard SLE treatments along with 200 mg/day DHEA supplements for up to 52 weeks; the result showed improved or stabilized signs and symptoms of disease, but adverse effects of acne and hirsutism were noted [79]. Another study also discovered the number of patients with flares and hospitalization decreased by 16% in those additionally receiving 200 mg/day DHEA. Yet, the study duration was relatively short, lasting only six months.

Healthy tubuloacinar epithelial cells possess the full intracrine machinery needed to process DHEA/DHEAS prohormones. Nevertheless, patients with Sjögren’s syndrome (SS) have low 3β- and 17β-hydroxysteroid dehydrogenases and subsequently low local DHT and androgen biomarker levels [116]. Failure in the intracrine conversion of DHEA within the affected tissue was observed in a former study, in which DHEA administration restored plasma androgen levels; however, it did not correct the local androgen deficiency in the salivary glands of patients with SS [117]. A 24-week randomized, double-blinded, pilot trial of oral DHEA (200 mg/day) supplementation was administered in patients in SS, and no significant improvement of hyposalivation or xerostomia was observed [118]. In a multicenter, investigator-led, randomized controlled trial, DHEA substitution at a dose of 50 mg/day was administered to patients with SS, and no significant improvement of fatigue symptoms was found [119]. Overall, no significant improvement of the symptoms in SS has been observed to date.

Biological sex has been identified as a significant predictor of remission in early RA [120] but the disease course tends to worsen more significantly in female patients [121]. The increased RA incidence is observed mainly in the elderly as androgen production declines with age. It has been suggested that aromatase CYP19 (estrogen synthase) polymorphisms [122] resulting in elevated enzyme expression or activity may lead to increased aromatization of androgen, and the synthesized estrogens are further converted to pro-proliferative estrogens, such as the 16-hydroxylated forms of estrone and 17β-estradiol [120,123]. This kind of effect would make the elderly more susceptible to RA. Under the circumstances, macrophage-like cells in the RA synovium are highly activated, which produce abundant cytokines and play an important role in the immune response in RA synovitis. The local immune-mediated interactions occurring in the adrenal gland may also lead to reduced androgen secretion. Lymphocytes and macrophages are both present in the zona reticularis, where these cells express MHC class II antigens, Fas, and Fas ligand [124]. Local cytokines, such as TGF-β and TNF-α, have been shown to suppress adrenal androgen production, including DHEA and DHEAS [122,125]. An RCT evaluated premenopausal women with rheumatoid arthritis who received 50 mg of DHEA daily for 12 weeks. The results showed a modest but favorable effect of DHEA compared with placebo in improving quality of life (QOL), including aspects such as physical health, psychological well-being, social relationships, and environment. Nevertheless, despite a reduction in ESR being observed in the DHEA group, the change was not statistically significant [126].

## 5. Discussion

The underlying cause of the symptoms in postmenopausal women is the decline of estradiol (E2) that results from the cessation of ovarian estrogen secretion at menopause, combined with the insufficient E2 formation from DHEA in the endometrium [35,127]. Intravaginal DHEA application improves vaginal pH, as well as epithelial cell counts, and relieves dyspareunia. In contrast, oral DHEA has not demonstrated significant improvement in menopausal symptoms while vaginal DHEA has shown benefits in women with vaginal atrophy [40]. Overall, previous studies have not shown significant benefits of systemic DHEA therapy comparing with placebo in terms of improving sexual function, well-being or metabolic health in postmenopausal women [128]. Recent studies have recommended DHEA as a treatment option for VVA in GSM [129,130,131,132]. A daily dose of 6.5 mg DHEA has been shown to improve VVA symptoms. Two phase III randomized, double-blind, placebo-controlled trials have also demonstrated that intravaginal DHEA application reduces vaginal pH, lowers the percentage of parabasal cells, and increases superficial cells. After 12 weeks of treatment, gynecological evaluations also show improved vaginal secretions, epithelial integrity, surface thickness, and color [130,133]. Intravaginal DHEA application also exhibits improvement in both urge urinary incontinence and stress urinary incontinence.

DHEAS does not directly bind to glucocorticoid receptors; contrarily, it may act as a functional antagonist to glucocorticoids [134,135]. This counterbalancing effect could explain the association between DHEAS levels and conditions linked to chronic cortisol elevation, such as cardiovascular disease, diabetes, and cognitive impairment [105,136].

When it comes to the relationship between endogenous DHEA and depression, it is suggested that the ratio of DHEA to cortisol levels plays an imperative role while encountering stress. Under stress, ACTH stimulates the adrenal cortex to release both cortisol and DHEA/DHEAS in the adrenal gland. A higher DHEA-to-cortisol ratio is thought to enhance resilience to stress. However, depression is a multifactorial disorder influenced by biological and social factors, and no definitive link between DHEA levels and depression has been firmly established.

Replacement with DHEA is not recommended to relieve hyposalivation or xerostomia in patients with SS [69]. On the other hand, DHEA has shown promising results in SLE, including stabilization of signs and symptoms and a reduced rate of flare-ups. These findings suggest potential therapeutic benefits, especially in patients with mild to moderate disease activity. No significance of improvement is found in patients with RA receiving DHEA treatment. The discrepancy between the effects of DHEA usage on SS, SLE, and RA is mainly attributed to their differing cytokine profiles. RA is predominantly driven by Th1 cytokines, including IL-2 and IFN-γ, while SLE is largely mediated by Th2 cytokines, such as IL-4, IL-5, IL-6, and IL-10 [137].

In almost all of the previous studies, side effects of DHEA were mild and did not require withdrawal from therapy, which were mainly based on androgenic side effects, such as hirsutism and acne. In summary, DHEA supplementation is usually safe, and its dosage can be adjusted according to individual health status and clinical situation. Current evidence has revealed that the therapeutic effects of DHEA supplementation are inconsistent in different human systems among different studies. The diversity of results is mainly due to heterogeneous receptor distribution, various action pathways, and distinct tissue responses in different systems. With regard to DHEA/DHEAS, the nature of the clinical effects on human systems is complex and tissue-specific, depending on the intracrine conversion and receptor subtype selectivity. Also, future research should focus on developing optimal dosing, long-term safety, patient selection criteria, and mechanistic biomarkers to clarify therapeutic potential across systems.

## 6. Conclusions

DHEA plays a crucial physiological role in aging and hormonal balance, particularly for postmenopausal women. Its conversion to sex steroids within target tissues offers a unique intracrine mechanism that may alleviate symptoms of GSM without systemic estrogen exposure. Intravaginal DHEA has demonstrated significant improvements in vaginal atrophy and urinary symptoms with minimal systemic effects. However, due to variability in clinical outcomes and limited large-scale data, cautious application and further rigorous studies are warranted to confirm long-term efficacy. On the other hand, DHEA and DHEAS play integral roles in maintaining central nervous system function, from regulating receptor activity to providing neuroprotection and influencing cognitive and emotional states. While experimental data support their benefits, clinical outcomes remain mixed, highlighting the need for further targeted studies to clarify their therapeutic potential in neurological disorders. Moreover, DHEA influences muscle, bone, cardiovascular, and immune health, particularly during aging. While it may enhance musculoskeletal strength, vascular function, and immune modulation, especially in inflammatory and autoimmune conditions, clinical outcomes vary. DHEA supplementation shows promise but should be approached cautiously, guided by further evidence from large, long-term clinical trials.
